# Small-Scale Intraspecific Life History Variation in Herbivorous Spider Mites (*Tetranychus pacificus*) Is Associated with Host Plant Cultivar

**DOI:** 10.1371/journal.pone.0072980

**Published:** 2013-09-13

**Authors:** Katherine Scranton, Menelaos Stavrinides, Nicholas J. Mills, Perry de Valpine

**Affiliations:** 1 Department of Environmental Science, Policy and Management, University of California, Berkeley, California, United States of America; 2 Ecology and Evolutionary Biology, Yale University, New Haven, Connecticut, United States of America; 3 Department of Agricultural Sciences, Biotechnology and Food Science, Cyprus University of Technology, Limassol, Cyprus; University of Toronto, Canada

## Abstract

Life history variation is a general feature of arthropod systems, but is rarely included in models of field or laboratory data. Most studies assume that local processes occur identically across individuals, ignoring any genetic or phenotypic variation in life history traits. In this study, we tested whether field populations of Pacific spider mites (*Tetranychus pacificus*) on grapevines (*Vitis vinifera*) display significant intraspecific life history variation associated with host plant cultivar. To address this question we collected individuals from sympatric vineyard populations where either Zinfandel or Chardonnay were grown. We then conducted a “common garden experiment” of mites on bean plants (*Phaseolus lunatus*) in the laboratory. Assay populations were sampled non-destructively with digital photography to quantify development times, survival, and reproductive rates. Two classes of models were fit to the data: standard generalized linear mixed models and a time-to-event model, common in survival analysis, that allowed for interval-censored data and hierarchical random effects. We found a significant effect of cultivar on development time in both GLMM and time-to-event analyses, a slight cultivar effect on juvenile survival, and no effect on reproductive rate. There were shorter development times and a trend towards higher juvenile survival in populations from Zinfandel vineyards compared to those from Chardonnay vineyards. Lines of the same species, originating from field populations on different host plant cultivars, expressed different development times and slightly different survival rates when reared on a common host plant in a common environment.

## Introduction

Life history variation is a characteristic feature of many natural systems [1]. The scale and drivers of variation may vary, with considerable consequences for population dynamics [2]–[4]. Variation at any scale may include phenotypic variation within a genotype, standing genetic variation within a population, and fixed differences between populations. Phenotypic or genetic differences between populations range from non-genetic polyphenisms to speciation [5] and can be driven by host plant use [6]. Many acarine mites, and spider mites (Family Tetranychidae) in particular, display significant amounts of life history variation and are prone to host-associated differentiation [7].

Numerous studies have shown rapid adaptation of spider mites to host plant species evinced by marked changes in life history processes such as survival, development and reproduction. Each of two isofemale lines of *Tetranychus urticae* adapted to different host plants had higher fitness and survivorship than the other line on its own “native” host plant [8]. Isofemale lines adapted to an unfavorable host plant also had higher survivorship than those adapted to a favorable host plant on novel marginal host plants. Populations of *T. urticae* adapted to unfavorable host plants (tomato and brocolli) experienced lower mortality, greater acceptance, and increased developmental rate (tomato only) than bean-adapted mites introduced to the unfavorable host plants [9]. *T. urticae* populations show adaptive plasticity in fecundity when comparing bean and tomato host plants [10]. Magalhaes et al. [11] found significant genetic variation and increases in juvenile survival and fecundity of *T. urticae* populations within 15 generations on novel hosts (tomato and pepper) and after 300 generations on cucumber host plants. Isofemale lines of *T. urticae* adapted to different host plants (tomato, Arabidopsis, and bean) varied in fecundity, in feeding damage on novel host plants, and differentially induced and responded to plant defenses [12].

Rapid adaptation to host plant species has also been demonstrated genetically, in addition to studies of ecological traits. Evidence from a genome-sequencing study of *T. urticae* showed that 24% of genes are differentially expressed upon host plant transfer from bean to a less favorable host plant (tomato or Arabidopsis), with the most profound changes occurring in genes in the detoxification and peptidase families [13]. Other genetic differences between spider mite lines adapted to different host plants have been found in micro satellite markers [14], allozyme and nuclear ribosomal sequences [15], and at the phosphoglucose isomerase locus [16]. Lines of the same species have become reproductively isolated, as has been reported for *T. urticae*, *T. kanzawai*, and *Oligonychus gotohi* [15], [17], [18]. Another herbivorous mite species, *Abacarus hystrix*, has also been shown to be reproductively isolated on different host plants [19]. The above studies clearly demonstrate both life history and genetic differences between isolated mite populations adapted to different plant species, but show no differences between populations that are not isolated or populations on host plants that are very similar.

Agricultural spider mite populations are not commonly isolated, instead situated in a contiguous landscape with a mosaic of suitable host plants. Individuals may encounter host plants across the landscape that vary widely from vegetables in gardens, to ornamental plants in residential landscaping, to crop plants in agricultural fields [20]. Spider mites disperse between host plants by crawling or by ballooning on a thread of silk carried by wind [21]. Aerial dispersal is usually less than 100 meters, but long distance dispersal events between 200 meters and 3 km have been reported [22]–[24]. The balance between the isolation caused by small scale dispersal and mixing from large scale dispersal events may determine the extent to which field populations adapt to host plant species or host plant cultivar.

Although host associated differentiation on different host plant species has been shown [7], it is less clear what differences to expect between populations on host plants of the same species or on different cultivars of the same species. The adaptive deme formation hypothesis suggests that herbivorous insects may adapt to the specific host plant, but experimental tests of the hypothesis have had mixed results [25]. A few studies have investigated local adaptation in mites on different cultivars or genotypes of the same species. An experimental manipulation of soil biota caused spider mites (*T. urticae*) on common bean plants (*Phaseolus vulgaris*) to adapt to the host conditions without any change of host plant species [26]. Herbivorous specialist mites (*Aceria parapopuli*) also showed local adaptation to host plant hybrid type [27]. Abundances of several species of herbivorous and predatory mites differed by cultivar (Merlot vs Verduzzo and Riesling vs Prosecco) in two *V. vinifera* vineyards about 3 ha large [28]. Levels of induced and constitutive resistance to *T. pacificus* were found to vary widely among six cultivars of *V. vinifera*, but the variation could not be clearly attributed to either the original host plant of the spider mites or to the phylogenetic relationships of the grape cultivars [29]. It is unclear whether specific morphological or physiological traits drive the differences in abundance and resistance, with little data to support such a hypothesis [30]. Variable resistance between cultivars has also been seen in spider mite herbivory on cotton [31], [32]. This evidence of resistance and abundance varying with cultivar suggests that life history traits vary with cultivar when isofemale lines are allowed to evolve in isolation.

Although previous studies have shown varying abundance of spider mites on cultivars in the field, and others have found life history differences between spider mite populations adapted to different host plant species, no study has yet explored the life history differences associated with adaptation to host plant cultivar. Our study investigates whether field populations of Pacific spider mites (*Tetranychus pacificus*) sampled from different cultivars of grapevine (*Vitis vinifera*) display significant life history differences on a common host plant. We evaluated whether development time, juvenile survival, and adult reproductive rate differ between field populations collected from Zinfandel and Chardonnay in a “common garden” experiment on lima bean plants (*Phaseous lunatus*) in the laboratory. We analyze the laboratory bioassay data with standard generalized linear mixed models and with time-to-event models common in survival analysis. We show that individuals from Zinfandel fields develop more quickly and may have higher juvenile survival than those from Chardonnay fields.

## Results

**Figure 1:**
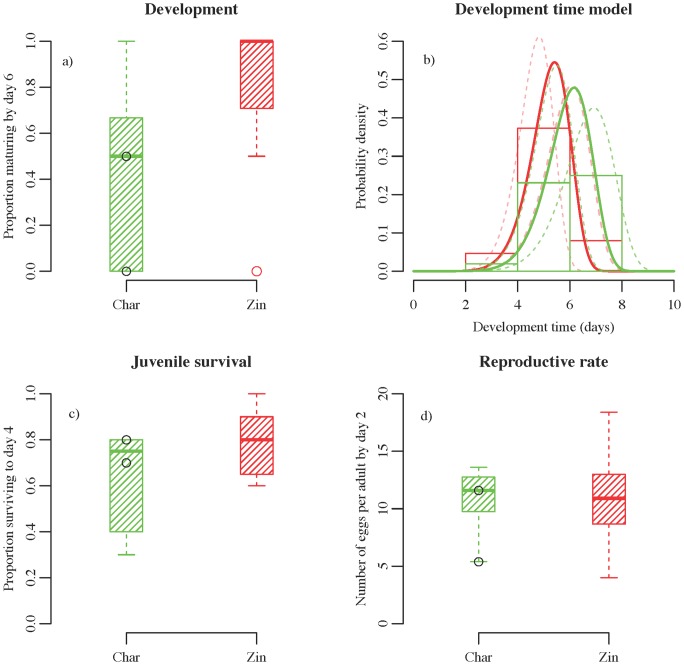
Results from the time-to-maturation model and generalized linear mixed model analysis. Throughout, data from Zinfandel source vineyards are in red, those from Chardonnay source vineyards are in green. Data values from two replicates of the Chardonnay vineyard that is further from the others are shown as black circles to illustrate the outlier test. a) Boxplot of data used for the GLMM of the probability of individuals maturing before day 6. b) Best-fit Weibull models for Zinfandel (solid red line) and Chardonnay (solid green line) source vineyards are shown, along with dashed lines that represent the extreme models under 0.05 and 0.95 quantiles of the estimated random effects. Models are plotted over histograms of the development data, separated by cultivar. c) Boxplot of data used for the GLMM of the probability of individuals surviving to day 4, d) Boxplot of data used for the GLMM of the number of eggs laid per mature adult female by day 2.

The developmental times of spider mite populations in our experiment differed significantly with source cultivar. The GLMM for the proportion of individuals matured after 6 days found a significant effect of source cultivar (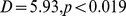 by randomization, 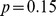 by large sample chi-squared approximation, Figure 1a).

The best-fit time-to-event model yielded a Weibull distribution for the development times of individuals from each cultivar (Figure 1b). Maximum likelihood estimates of the Weibull parameters revealed that populations founded from mites collected from Zinfandel developed more quickly than those collected from Chardonnay (shape: 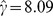, rate: 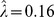, fixed cultivar effect coefficient: 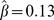). Likelihood ratio tests showed that the fixed cultivar effect was significant in the mixed-effects Weibull time-to-event model (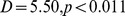 by randomization, 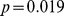 by large sample chi-squared approximation). The estimated random effects show that assay populations varied randomly in development time, but different sample colonies did not. Maximum likelihood estimates of the standard deviation of each random effect revealed moderate variation between assay populations (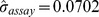) and zero variation between sample colonies (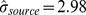
×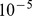). Figure 1b shows the range of variation due to these random effects with dashed lines depicting lower and upper bounds for the estimated Weibull distribution.

The probability of survival of juveniles after 4 days was, on average, 0.767 for Zinfandel and 0.633 for Chardonnay. Analysis with a GLMM found that this difference was marginally significant 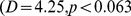, by randomization, 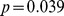, by large sample chi-squared approximation, Figure 1c). Females laid on average 10.8 eggs by day two of the assay, independent of source cultivar 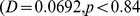, by randomization, 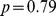, by large sample chi-squared approximation, Figure 1d).

**Figure 2:**
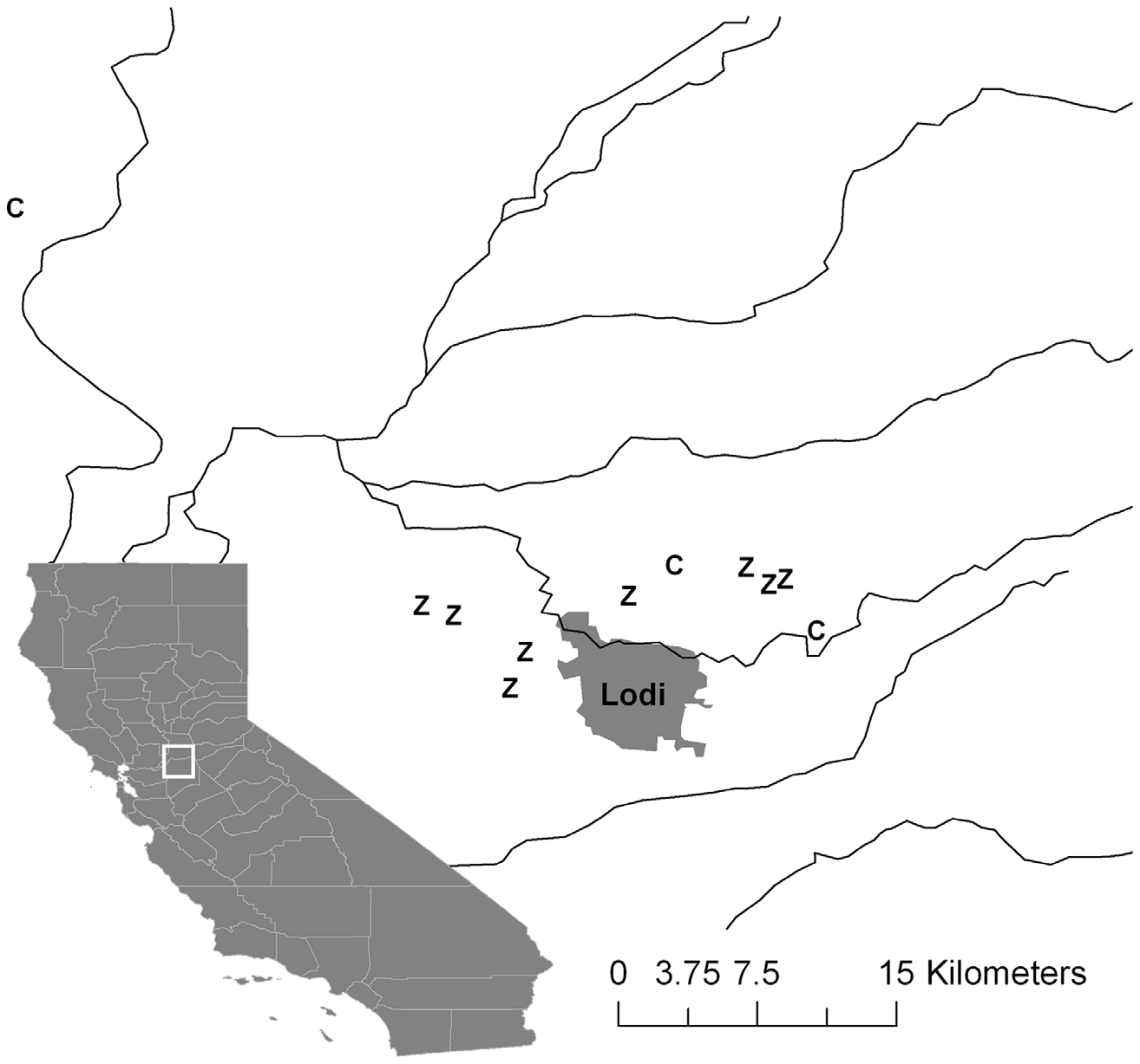
Map of sampled vineyards in San Joaquin, Sacramento and Yolo Counties, surrounding the town of Lodi, California. Rivers are shown as solid lines. “C” markers denote fields of Chardonnay grapes and “Z” markers denote Zinfandel. The white square on the state map shows the location of our sites within California.

Since one Chardonnay site was geographically farther than the other sites were to each other, we examined whether it represented an outlier for any analyses. Plotted data values (Figure 2) show that the for all life history traits the far site yielded data similar to the other Chardonnay sites. The data values are not extreme and fall within the range of values from all other assay populations.

## Discussion

Our study has demonstrated significant differences in life history parameters in populations of Pacific mite associated with host plant cultivar. When reared on a common, favorable host plant, mites from a source population on Zinfandel grapes matured more quickly and likely experienced higher juvenile survival than individuals from Chardonnay source populations. The distribution of the vineyards, the dominance of vineyards in the area, and the capacity for long range dispersal of the mites indicates that source populations were not geographically isolated. Vineyards are distributed in a mosaic across the landscape with the possibility for movement of individuals between them.

The life history differences we found are supported by the few previous studies comparing spider mite abundance on different cultivars. English-Loeb et al. [29] report slightly higher *T. pacificus* abundance on Zinfandel as compared to Chardonnay, which may be explained by the shorter development time and greater juvenile survival observed in our study. Both shorter development time and greater juvenile survival would independently lead to a higher intrinsic rate of increase as reported for spider mites by Helle and Sabelis [20].

The local adaptation to host cultivar that we found in our study may have several potential causes. Given the tendency of spider mites to form host races, our results may be an indication of host race formation on the two different cultivars. The results may also indicate adaptive deme formation, where some characteristic of the cultivar drives responses in life history traits. Numerous studies have found that tetranychid mites respond to selection on life history traits rapidly, within 6–15 generations [9], [33], [34]. Adaptive phenotypic plasticity and host-associated differentiation can lead to evolutionary and ecological changes [31], [35] and sympatric speciation is common among phytophagous insects [36]. However, our study did not attempt to address any evolutionary aspects of the cultivar-associated life history differences such as the timescale of adaptation to cultivar, the potential for speciation or the amount of gene flow between field populations. We also did not investigate possible physiological or morphological traits of the cultivars that may have been driving the selection for life history traits. Leaf hair density and other leaf characteristics have been shown to affect predatory mite density by affecting habitat quality, but studies have not shown the same effect for herbivorous mites [30]. The capacity of the spider mite for both ambulatory and long-range dispersal, as well as the complex spatial structure of the grapevine, likely play a large role in local adaptation, suggesting many avenues of future research.

Our analysis of the cultivar-associated life history differences incorporated the use of a mixed-effects time-to-event model that provided additional detail and biological realism compared to standard GLMMs. Time-to-event models yielded information about the rate and shape of the distribution of development times over the entire time span of the study. The fitted model gave us insight into times at which individuals are most likely to mature and how likely individuals are to mature at the extremes, either very early or very late. The time-to-event model also utilizes information from every sample date, whereas a GLMM estimates binomial probability of survival or death at only one sample date. Multiple GLMMs could yield survival estimates at multiple sample dates, but this would increase the number of parameters to estimate and lead to the potential introduction of type 1 errors, depending on the number of hypothesis tests involved.

Photographic sampling is common in observational studies of other species using camera traps, remote sensing or other aerial data. Most studies on herbivory that employ a photographic sampling scheme either destructively sample leaves, or quantify leaf damage, not herbivore abundance. A notable exception is a well-established non-destructive photographic sampling method for whiteflies [37]–[40]. Our methods were limited by the difficulties of creating and testing feasible algorithms for automated counts. One main challenge is the lack of color contrast between the spider mite and lima bean leaf. Other ecological systems and advances in image processing and object-based image analysis may allow future studies to employ fully or semi-automated counting of individuals.

The scope of our study was also limited to populations on a common laboratory host plant (*Phaseolus lunatus*); we did not attempt to follow populations on the native grape cultivar. Nonetheless, pest management decisions could be affected depending on the degree to which our findings translate to field populations on native host plants. Pest managers and growers weigh the abundance, the speed of development (mostly relying on temperature as a proxy), and the harvest time of the crop against the cost of pesticide applications or release of natural enemies. Thus, differences in development time and survival in field populations among grape cultivars could have important impacts on the decision to apply miticide and the timing of application. Our results may directly affect the way growers think about the speed of spider mite development in different vineyards.

Our results also highlight the need to investigate the impact of life history variation on population growth rate of spider mite populations. Some empirical studies have found host-associated differences between spider mite populations in the intrinsic rate of increase [41] and theoretical studies predict strong effects of life history variation on population dynamics [2], [42]. Our study was limited to specific demographic traits, not any metric of overall dynamics, but by establishing the presence of life history differences in sympatric populations on very similar host plants we have hopefully highlighted the importance of investigating the effects of life history variation on dynamics. A central tenet of population ecology is the importance of understanding the drivers of population dynamics and in many cases life history variation merits consideration as one of those drivers.

## Materials and Methods

The Pacific spider mite (*T. pacificus*) is an herbivorous arthropod pest in many agricultural systems, including the vineyards of California's central valley. This phytophagous mite feeds by piercing leaf cells with its mouthparts and sucking out the cell contents [20]. *T. pacificus* has 5 distinct life stages including one larval stage and two nymphal stages (protonymph and deutonymph) and undergoes a quiescent period at each stage transition. Its demography (including development rate and fecundity) is highly temperature sensitive [43], [44] and highly variable [45].

*T. pacificus* were collected from privately owned vineyards (*Vitis vinifera*) near Lodi, CA in the San Joaquin Valley from mid-July through late-August 2009 (Figure 2). All sampling was done in the presence of pest control managers hired by the growers with explicit approval to sample pests and manage infestations. The study site lies in California grape crush district 11, which had 69,220 total standing acres of wine grapes (in 2009) including Zinfandel (18,800 acres), Chardonnay (13,563 acres), Cabernet Sauvignon (11,272 acres), and Merlot (7,497 acres) [46]. Fields of single cultivars are distributed across San Joaquin and Sacramento counties and are often adjacent to different cultivars.

Mites were sampled from two different cultivars of grapevine: Zinfandel and Chardonnay, which are the most common cultivars in our region [46], favorable hosts for the Pacific spider mite [29], and cultivated the same way by growers in terms of water, fertilizer and pesticide applications. Possible sites were chosen through discussions with local farm advisors and pest control advisors, however sampling was hindered by the prompt treatment of outbreaks with acaricide and by cooler than normal temperatures. Although *T. pacificus* occurs regularly on Chardonnay, during the year of our study outbreaks were rare and quickly treated by growers, which limited our sample size. Outbreaks that did occur were sampled at the initial stages of population expansion, as soon as spider mites were reported. Over the sampling period, mites were collected from 8 Zinfandel vineyards and 3 Chardonnay vineyards. At each site 10–15 leaves were clipped from infested grapevines and placed in paper bags labeled with identifying information. Sample bags were transported back to the laboratory in a cooler at 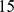
°C.

Field samples were processed at the Oxford Tract Greenhouse and Insectary at UC Berkeley. If samples were not processed on the same day as collection, they were transferred to sealed Tupperware containers with moistened paper towels and kept in an incubator at 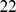
°C with a 16L : 8D h photoperiod for less than 24 hours. 50–100 mated adult females were transferred from samples to greenhouse-grown, uninfested bean plants (*Phaseolus lunatus*) to found a *sample colony*.

All sample colonies were kept in a growth room on a 16L : 8D h photoperiod at 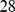
°C constant temperature and 36% relative humidity (RH). Each sample colony was maintained on 2 large bean plants in a 0.6×0.6×0.6 meter cage on an elevated rack under full-spectrum fluorescent grow lights. Cages were made of plexiglass with ventilation on two sides and on the top, made of 156 grade mesh with openings fine enough to prevent movement of mites, and sealed tightly at edges with plexiglass glue. Door edges and hinges were sealed with duct tape and precautions were taken to prevent mite movement when cage doors were opened to water plants. Cages were elevated on overturned pots, whose bases were ringed with Stikem (Seabright Laboratories, Emeryville, California) to prevent mites from climbing up the pots. Fresh plants were rotated in after 7–10 days to provide extra habitat and promote mite population growth. Sample colonies were maintained for 14–21 days. The actual time that individuals spent on bean plants was long enough to discount any maternal effects from field conditions and short enough to prevent adaptation to the lima bean host plant. All field samples were processed using the same procedures and there were no systematic differences between cultivars in field collection date or assay date.

Individuals from each sample colony were used to found 4 *assay populations*. Two assay populations were initiated with a cohort of 10 eggs and two with a cohort of 5 mated adult females. Egg cohorts were used to gather information on egg and juvenile survival and stage duration, while adult cohorts were used to study reproductive rate and adult survival. Egg cohorts were initiated by directly transferring 10 nearly hatched eggs (with visible eyespots) to the assay population host plant with a paintbrush. To obtain an adult cohort of 5 newly-emerged, mated adult females, we removed approximately 10 females in the third (final) quiescent stage and 10 adult males from the sample colony and placed them on a large leaf disc surrounded by damp cotton in a petri dish. Adult males guard quiescent females and mating occurs as adult females emerge [20]. After 24 hours we transferred 5 adult females that had emerged and mated to a new, clean leaf disc and pinned the disc to the assay population host plant.

Each assay population host plant was a potted bean plant approximately 3 weeks old. Plants were washed to protect against pest infestation, repotted, and trimmed to two large, paired leaves each. Leaves were trimmed to flat rectangular sections approximately 6 cm by 4 cm to control leaf surface area across all assay populations and to allow for clearly focused images. Trimmed, repotted plants were kept under grow lights in the growth room for 1–3 days before use to ensure they were healthy and free of pests.

Each assay population host plant sat in a small tray in one square of a large grid of Stikem. Mite movement was further prevented by Stikem around the lip of the tray and the top edge of the pot. No mites were noted in the Stikem over the course of the experiment. Assay populations were maintained for 12 days under grow lights in the same growth room (16L : 8D h photoperiod at 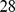
°C 36% RH) and temperature recordings were kept. Temperature recordings showed reliably constant temperatures, with slight fluctuations within a 2.8 degree range around the average temperature 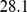
°C. Average hourly change in temperature was less than 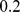
°C. Temperature was not a significant factor in any of the analyses.

We chose to follow the assay populations in situ with a non-destructive sampling scheme using photography. Destructive photographic sampling of single leaves cut from a plant or tree is commonly used to quantify leaf damage by herbivory [47], [48]. Destructive photographic sampling has also been used to estimate spider mite abundance directly [49]. Non-destructive photographic sampling of herbivores in situ is difficult because of our inability to physically manipulate the leaf due to the risk of disturbing or injuring individuals, but has been done for beech scale on bark (*Crytococcus fagisuga*) [50] and for whiteflies on leaves (*Bemisia argentifolii* and *Aleurocanthus woglumi*) [37], [39], [40].

We sampled leaves in situ with a digital camera (Nikon D5000) and 60 mm macro lens without touching the leaves. Every 48 hours, photographs were taken of each assay population. Four images (1 per quadrant) were taken of each side of each leaf for each assay population with 2 leaves. This summed to 16 images per assay population per sample date. Mites were counted by eye with the aid of basic MATLAB image analysis and data organization tools. Individuals were categorized as eggs, immatures, or adults, and resulting data sets followed population abundance for each stage for 12 days.

### Analysis

The common garden experiment yielded 44 assay populations: two egg cohorts and two adult cohorts from each of 11 sample colonies. Each of the 44 assay populations yielded a data set that tracked the abundance of a cohort over 12 days. Sampling was conducted at two day intervals, yielding 7 samples per assay population. We focused on development time and juvenile survival probability from the 22 egg-initiated assay populations and on reproductive rate from the 22 adult-initiated assay populations. The goal of our analyses was to detect any potential differences in these three life history processes between assay populations from Zinfandel and Chardonnay vineyards.

To estimate development time from egg-initiated assay populations, we used the number of adults on each sample date. If the number of adults increased between sample dates, we inferred that immature individuals had developed into adults during the interval between sample dates. Thus for each assay population, we created a count of individuals maturing during each sample interval. Our data is *interval-censored*, where the two sample dates create a lower bound and upper bound for the exact maturation time which remains unknown.

We first analyzed the development data with a generalized linear mixed model (GLMM). For the GLMM, we condensed the development intervals by grouping individuals into those that matured before day 6 and those that matured after day 6. We used a binomial regression to estimate the effect of cultivar on the probability of maturing to adulthood before day 6, with two random effects for sample colony and assay population and a fixed cultivar effect. We also used a second approach in analyzing the development interval data: a mixed-effect time-to-event model that allows incorporation of all sample intervals.

Time-to-event models are a class of models from survival analysis with an underlying assumption of individual heterogeneity in the population: individuals experience the event at slightly different times, according to a probability density function [51]. The specific probability density function can be chosen to fulfill specific assumptions in the data or to allow for a flexible shape in the probability of an individual experiencing the event. Time-to-event models can be extended to include covariates and random effects, or variation between groups [51]. Models that include random effects (frailty models) stem from the idea that unknown, inherent traits would cause an individual to be more or less frail (in our case more or less likely to mature) and could be shared between members of a group (in our case an assay population or a sample colony) [52].

We fit a mixed effects time-to-event model to development times using a Weibull distribution to describe the probability of maturing as an individual ages, similar to the model used by Bellamy et al. [53].
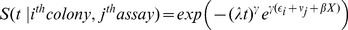
(1)Where 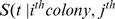 assay) is the probability of an individual, in the 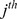 assay population from the 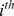 sample colony, had a development time greater than 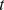. The Weibull distribution is commonly used in survival analysis models and had two parameters: a shape parameter (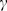), and a rate parameter (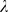) that was affected by the random effects and the fixed cultivar effect (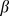) on a log scale. We included two random effects for sample colony (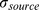) and assay population (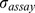) and assume 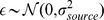 and 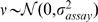. These hierarchical random effects, along with the interval-censored data, made the likelihood function complex. Since there is no widely available software for mixed-effect time-to-event models with interval censored data we programmed likelihood calculations directly in R [54] using code from Scranton and de Valpine [55]. We estimated the Weibull parameters (shape, 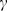, rate: 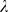) and tested the significance of the fixed cultivar effect (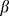, the difference between cultivar sources in log lambda) while accounting for the two sources of random variation.

To estimate juvenile survival probabilities from the egg-initiated assay populations we used the counts from the photographs of juveniles present on day 4 out of the initial 10 eggs in an egg-initiated assay population. Choosing day 4 struck a balance between allowing individuals sufficient time to experience any possible mortality while still ensuring that none of the individuals would have yet matured. After day 4, we could not confidently attribute changes in the number of juveniles to juvenile mortality. Individuals may have matured and then died. New eggs entering the population also started to mature, complicating the task of classifying the fate of original members of the cohort. Using only two intervals of event times ([0,2], [2], [4]) made a time-to-event approach unlikely to provide more useful information than a single survival rate. We used a binomial regression to estimate the effect of cultivar on the probability of surviving to day 4, with random effects for the sample colony and for the replicate assay populations, a fixed cultivar effect and no other factors.

To estimate reproductive rates from the adult-initiated assay populations, we counted the number of eggs present on day two. We used the number of surviving adult females on day two as a fixed effect to account for adults that may have died before reproducing. We again included the cultivar as a fixed effect and two random effects for the sample colony and for the replicate assay populations in a GLMM. We used a poisson regression for egg counts, the appropriate model for count data, with the two random effects and two fixed effects mentioned above. All GLMM analyses were done in R using the lme4 package, version 0.999375-34, [56].

For both the time-to-event model and the GLMMs, we fit models with and without the (null) cultivar effect and assessed the significance of the fixed effect with likelihood ratio tests. These tests use large sample approximations to the chi-squared distribution, but we had concerns about our small sample sizes so we also performed randomization tests. We randomized the dataset by cultivar 1000 times to create a null distribution for each of the likelihood ratio test statistics (D), on which we base our main conclusions.
